# Comparison of Vitamin E and L-Carnitine, Separately or in Combination in Patients With Intradialytic Complications

**DOI:** 10.5812/numonthly.10670

**Published:** 2013-07-07

**Authors:** Hamid Tayebi Khosroshahi, Bohlul Habibi Asl, Afshin Habibzadeh, Parastoo Chaichi, Amin Ghanbarpour, Amir Hossein Badie

**Affiliations:** 1 Department of Nephrology, Liver and Gastrointestinal Disease Research Center, Tabriz University of Medical Sciences, Tabriz, IR Iran; 2 Department of Pharmacology, Tabriz University of Medical Sciences, Tabriz, IR Iran; 3 Department of Cardiology, Cardiovascular Research Center, Tabriz University of Medical Sciences, Tabriz, IR Iran; 4 Medical Philosophy and History Research Center, Tabriz University of Medical Sciences, Tabriz, IR Iran

**Keywords:** Renal Dialysis, Kidney Failure, Chronic, Vitamin E, Carnitine

## Abstract

**Background:**

The most common complications during dialysis are hypotension and muscle cramps. There are many strategies to prevent and treat these complications.

**Objectives:**

The aim of this study is to evaluate effects of vitamin E and L-carnitine supplementation alone and in combination on intradialytic complications.

**Patients and Methods:**

In a prospective study, 20 patients with end stage renal disease on chronic hemodialysis that had intradialytic complications such as hypotension, muscle cramp, nausea, vomiting and headache were studied. These patients were studied in four 45 day periods, beginning with no treatment (step 1), receiving vitamin E (200 IU/d) (step 2), receiving L-carnitine (500 mg/d) (step 3) and their combination (step 4). Intradialytic complications were recorded in each step and compared between treatments.

**Results:**

All three treatments significantly reduced frequency of muscle cramps in comparison to baseline values. Vitamin E alone and in combination with L-carnitine reduced the frequency of muscle cramps more effectively. Hypotension was significantly lower in combination therapy in comparison to baseline values and vitamin E treatment.

**Conclusions:**

Vitamin E and L-carnitine both have comparative effects on intradialytic complications. As the combination use of vitamin E and L-carnitine could more effectively reduce the intradialytic complications, it is recommended for daily use in hemodialysis patients.

## 1. Background

The prevalence and incidence of chronic renal failure (CRF) is increasing all over the world, and this has become a serious problem. CRF is characterized by slow and progressive decline in the kidney function (1). Hemodialysis, a lifesaving procedure for advanced CRF patients, is associated with a number of acute intradialytic complications and symptoms such as hypotension, chest pain, back pain, muscle cramps, nausea, vomiting, headache, pruritus and fatigue (2-4).

Intradialytic hypotension is a common complication with associated symptoms such as nausea, vomiting, dizziness and cramps. Intradialytic hypotension may cause premature discontinuation of hemodialysis that may lead to chronic underdialysis and higher mortality risks (5, 6). The other prevalent complication is muscle cramp which causes severe pain and interferes with normal life function (7-9).

Various treatments are proposed, amongst them vitamin E and L-carnitine supplementation are the most beneficiary ones. As vitamin E reduces oxidative stress, many studies have tested vitamin E supplementation as a therapy to prevent various chronic diseases. Vitamin E has been recommended as the initial treatment of choice for HD cramps (10, 11).

L-carnitine is a quaternary amine known to transfer long chain fatty acids from cytoplasm into the mitochondrial matrix to be oxidized (12). In humans, carnitine plays a pivotal role in energy metabolism (13). There is marked reduction in the production of carnitine in end-stage renal disease (ESRD) patients, and is significantly reduced in hemodialysis patients (14, 15). Regular carnitine supplementation in hemodialysis patients may reduce the incidence of intradialytic muscle cramps, hypotension, asthenia, muscle weakness, and cardiomyopathy (16-19); however, its effects on dialysis-related muscle cramping or intradialytic hypotension are questioned by another study (20).

## 2. Objectives

Although there are various reports considering vitamin E and L-carnitine effects on intradialytic complications, there is no study comparing the effects of these two supplements. In this study we aim to evaluate that which treatment including vitamin E, L-carnitine alone or in combination has more beneficiary effects.

## 3. Patients and Methods

### 3.1. Study Population

In this prospective study, we evaluated the effect of L-carnitine, vitamin E or their combination supplementation on intradialytic complications. For this purpose, 20 adult patients (11 male, 9 female with mean age of 51.35 ± 12.90 years) who had been undergoing thrice-weekly HD treatments, each time for 4 hours for at least 1 year at 2 facilities (Sahid Madani hospital and Sina hospital) with an arteriovenous fistula as blood access and anuria with no renal function for a minimum of 1 year were included. The mean blood flow of 350 mL/min and bicarbonate dialysate flow rate of 500 mL/min were maintained during dialysis. All these patients had complaints about intradialytic complications in the 3 months preceding enrollment. Subjects who had cardiovascular disease with Class III and IV symptoms, as defined by the New York heart association criteria, were excluded. The ethics committee of Tabriz university of medical sciences approved the study, and all subjects gave informed consent.

### 3.2. Study Design

Patients were scheduled to receive various treatment protocols for 6 months. Patients were first evaluated for 45 days (10 dialysis sessions) without any treatment as baseline and their complications during dialysis were recorded (step 1). Then patients were given oral vitamin E supplementation (200 U/daily) for the next 45 days (step 2). For the next 45 days, vitamin E supplement was stopped and patients were given oral L-carnitine (500 mg/daily) (step 3). During the last 45 days, patients were given vitamin E supplementation (200 U/daily) and oral L-carnitine (500 mg/daily) together (step 4). During the study period, patients were advised to maintain their everyday diet, medication or daily activities. Those who could not keep up with the study protocol or with drug intolerance were excluded.

Any complications during dialysis including muscle cramp, pain and nausea and vomiting were recorded. The occurrence of painful muscle cramps was recorded. A muscle cramp was defined as a painful involuntary muscle contraction that lasted more than 1 min during the dialysis (7). Blood pressure was measured every 30 min. An intradialytic hypotensive episode was a decrease of more than 15 mmHg in either systolic or diastolic blood pressure.

### 3.3. Statistical Analyses

Statistical analyses were performed using the statistical package for social sciences, version 16.0 (SPSS, Chicago, Illinois). Continuous values were expressed as mean ± standard deviation and categorical variables were expressed as percentages. The categorical parameters were compared by Chi-square tests or Fisher’s exact test. A p value < 0.05 was considered significant.

## 4. Results

Cause of ESRD was diabetes in 8 cases, glomerulopathy in 7 cases, tubular disease in 3 cases and with unknown etiology in 2 cases.

Patients' complications during dialysis are shown in Table 1. There was a significant difference in frequency of muscle cramps between steps, but the difference in other complications was not significant. 

Muscle cramps: All three treatments significantly reduced frequency of muscle cramps in comparison to baseline values (P < 0.001 for vitamin E and vitamin E and L-carnitine combination; P = 0.03 for L-carnitine). The combination treatment (vitamin E and L-carnitine) had significantly higher reduction in frequency of muscle cramps than L-carnitine (P < 0.001), but the difference with vitamin E alone was not significant (P = 0.07). Vitamin E in comparison to L-carnitine reduced more effectively the frequency of muscle cramps (P = 0.006) (Table 1). 

**Table 1.** Intradialytic Complications in Different Treatment Protocols

	Step 1 (No Treatment)	Step 2 (Vitamin E)	Step 3 (L-Carnitine)	Step 4 (Vitamin E and L-Carnitine)	P value
**Frequency of muscle cramp **	6.40 ± 1.63	3.85 ± 1.42	5.25 ± 1.61	3.15 ± 0.93	P < 0.001
**Hypotension, No.(%)**	16 (80)	16 (80)	14 (70)	9 (45)	0.055
**Nausea and vomiting, No.(%)**	6 (30%)	5 (25)	5 (25)	4 (20)	0.91
**Chest and/or back pain, No.(%)**	5 (25)	4 (20)	4 (20)	4 (20)	0.97
**Pruritus No.(%)**	2 (10)	2 (10)	1 (5)	1 (5)	0.86

Hypotension: In comparison to baseline findings, hypotension was significantly reduced with combined treatment (vitamin E and L-carnitine) (P = 0.04), and not with vitamin E or L-carnitine alone. There was also significant reduction in combined treatment in comparison to patients receiving vitamin E (P = 0.04); but the difference between patients receiving L-carnitine and combined treatment was not significant (P > 0.05) (Table 1).

Other complications: There was no significant reduction of neither nausea and vomiting, nor pain and pruritus by any of treatments (P > 0.05) (Table 1). No supplement related adverse effects were encountered during the trial.

## 5. Discussion

Chronic Renal failure is a common public health problem, which occurs in many countries with an increasing prevalence. Hemodialysis, which is one of the renal replacement therapies, is a life-saving treatment. However, hemodialysis is accompanied by several complications. The most prevalent complications are muscle cramp and hypotension. Other common complications include nausea, vomiting, headache and pruritus (21-25). As these complications have adverse effects on the quality of life of the patients, there are efforts to reduce them and improve patients' quality of life. 

Vitamin E and L-carnitine are the two supplements evaluated in this respect which have shown beneficiary effects. It seems that L-carnitine administration might be rational in hemodialysis patients as it may improve several situations, such as cardiac performance, as well as positive protein balance induction, insulin resistance reduction, and chronic inflammation amelioration (26).

We found no study evaluating the combination of these treatments. The combination of vitamin E and L-carnitine are studied in different studies and have shown better results in comparison to separate use of vitamin E and L-carnitine (27-29). In this study we evaluated the effect of vitamin E and L-carnitine alone and in combination in reducing intradialytic complications.

Muscle cramps are the most prevalent intradialytic complication and most studies have tried to introduce palliative treatments to it. We observed a significant reduction in frequency of muscle cramps in all three treatment protocols in comparison to baseline values. Vitamin E alone or in combination also had a more significant reduction in frequency of muscle cramps than L-carnitine supplement. El-Hennawy and Zaib (10) also reported reduction of cramps attack after vitamin E supplementation. The effects of L-carnitine on relieving muscle cramps are shown in various studies, as well (16, 17, 19). Khajedehi and colleagues (30) also reported better results in patients receiving vitamin E and combination of vitamin E and vitamin C in reducing muscle cramps. Our results and Khajedehi and colleagues' (30) are indicative of better effects of vitamin E in combination therapy.

Ahmad et al. (16) reported a reduction in hypotension rate in patients receiving L-carnitine. However, a meta-analysis by Lynch and colleagues (20) could not confirm a beneficial effect of L-carnitine supplementation on dialysis-related intradialytic hypotension. We found no study evaluating the effects of vitamin E supplementation on intradialytic hypotension, although it is shown that vitamin E-modified dialyzers could reduce hypotension (31). In this study we observed no beneficiary effect of vitamin E or L-carnitine alone on intradialytic hypotension. However, hypotension was significantly reduced with combined treatment of vitamin E and L-carnitine.

We found no significant reduction of neither nausea and vomiting, nor pain and pruritus by any of treatments; this could be due to the low frequency of these complications at the beginning of the study.

There is limited data on the efficacy of oral L-carnitine and the use of oral supplements due to its limited bioavailability, high doses of drug (32) and probable side effects of its toxic metabolites (33, 34) is not recommended. The reported usually used oral dose of L-carnitine in previous studies was 1000-3000 mg/d (35), unlike our study (500 mg/d). The lower effects of L-carnitine in reducing intradialytic complications could be due to lower dose of L-carnitine administered in our study. Similar to our findings, Debska-Slizien and colleagues (36) observed that L-carnitine in low doses (500 mg/d) can improve plasma total and free carnitine levels; this improvement subsequently could improve some markers, indicative of effectiveness of low dose L-carnitine supplementation. However, due to its better results in low dose in combination with vitamin E with no probable complications of its use, L-carnitine could be recommended for combination treatment of intradialytic symptoms.

Conclusion: Both vitamin E and L-carnitine supplements have good and comparative effects on improving intradialytic complications. The combination of vitamin E and L-carnitine combination was more effective than treatments alone and could more effectively reduce the intradialytic complications and so it is recommended for daily use in hemodialysis patients.
